# Study on the Calculation Models of Bus Delay at Bays Using Queueing Theory and Markov Chain

**DOI:** 10.1155/2015/750304

**Published:** 2015-02-11

**Authors:** Feng Sun, Li Sun, Shao-wei Sun, Dian-hai Wang

**Affiliations:** ^1^College of Transportation, Shandong University of Technology, Zibo 255049, China; ^2^College of Civil Engineering and Architecture, Zhejiang University, Hangzhou 310058, China

## Abstract

Traffic congestion at bus bays has decreased the service efficiency of public transit seriously in China, so it is crucial to systematically study its theory and methods. However, the existing studies lack theoretical model on computing efficiency. Therefore, the calculation models of bus delay at bays are studied. Firstly, the process that buses are delayed at bays is analyzed, and it was found that the delay can be divided into entering delay and exiting delay. Secondly, the queueing models of bus bays are formed, and the equilibrium distribution functions are proposed by applying the embedded Markov chain to the traditional model of queuing theory in the steady state; then the calculation models of entering delay are derived at bays. Thirdly, the exiting delay is studied by using the queueing theory and the gap acceptance theory. Finally, the proposed models are validated using field-measured data, and then the influencing factors are discussed. With these models the delay is easily assessed knowing the characteristics of the dwell time distribution and traffic volume at the curb lane in different locations and different periods. It can provide basis for the efficiency evaluation of bus bays.

## 1. Introduction

In recent years, with the rapid development of public transport, bus bays face an increasing pressure especially during peak hours. While serving passengers at a bus stop, buses can interact in ways that limit their discharge flows. This can increase bus delay at bays and degrade the bus system's overall service quality [1–3]. So it is necessary to evaluate the operating efficiency of bus bays and analyze the reasons for the increase of bus delay and then put forward the countermeasures to reduce the delay.

Though professional handbooks [4, 5] have long offered formulas and tables for estimating bus-stop discharge flow, these are known to be unreliable [3, 6]. The existing studies are mainly empirical formula based on the statistical analysis of actual survey data [7–10] and lack of theoretical model on computing efficiency. Therefore this paper studied the calculation models of bus delay at bays based on the analysis of the bus operating characteristics.

## 2. Analysis of Bus Delay at Bays

**Figure 1:**
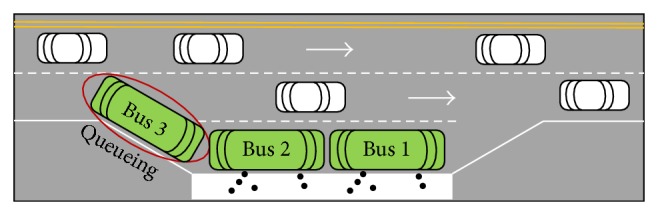
The behavior of queueing for serving at bus bays.

The form of bus bay is shown in Figure 1.

The berths are numbered 1 and 2 from the front to the back, and three buses arriving at the bus bay are numbered 1, 2, and 3 according to the arrival sequence. When buses 1 and 2 occupied berths 1 and 2 to serve their passengers, bus 3 must queue for entering upstream of the stop, as shown in Figure 1. The waiting time during this process is called entering delay.

**Figure 2:**
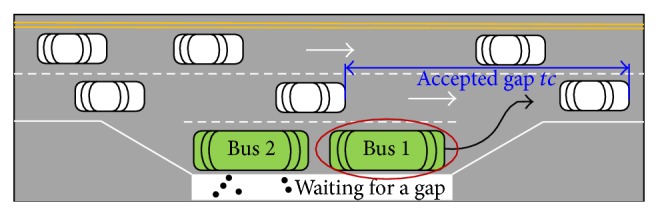
The behavior of waiting for a gap at bus bays.

In addition, when the serving is over at bus bays, the driver must look for a safe opportunity or “gap” in the traffic flow of curb lane to join them, as shown in Figure 2. The waiting time during this process is called exiting delay.

Therefore, the bus delays at bays mainly including entering delay and exiting delay are computed by the following equation:

(1) $$\begin{array}{c} W=W_{q}+W_{b}, \end{array}$$

where *W*_*q*_ is the entering delay, s; *W*_*b*_ is the exiting delay, s.

We next derived the calculation models of bus entering delay and then studied the computing models of bus exiting delay. The calculation models of bus delay at single-berth and two-berth bays are proposed, respectively, finally.

## 3. Calculation Models of Bus Entering Delay

### 3.1. Queueing Model of Bus Bays

At the bus bays serving some lines, buses enter the berth sequentially, then load and unload passengers, and finally exit the stop. So the buses and the stop constitute a queuing system [11]. According to the present study, buses arrived at the stop as a Poisson process, as may occur when a moderately busy stop serves multiple bus routes [12, 13]. So a stop with a general service time distribution and *c* berths can be denoted as the *M*/*G*/*c* queueing system. In this system, we define a regenerative point as the instant in time when the buses in all berths have discharged from the stop [14]. (Though the stop is empty at each regenerative point, it may be filled immediately thereafter if a bus queue is present at the entrance.) The time interval between two successive regenerative points is defined as a cycle. Let *L*_*n*_ be the number of buses queued at the stop's entrance at the beginning of the *n*th cycle (i.e., the *n*th regenerative point); *λ* is the rate of Poisson bus arrivals; and recall that *c* is the stop's number of serial berths. So we claim that the stochastic process {*L*_*n*_} is a Markov chain in given *λ*, *c*, and the distribution of bus service times at the stop. A Markov chain is a sequence of random variables *X*_1_, *X*_2_, *X*_3_, … with the Markov property, namely, that, given the present state, the future and past states are independent. In this system, the average bus delay in queue can be calculated once the Markov chain limiting probabilities are identified.

Based on observations of bus operations in China, three assumptions are adopted in the course of formula derivation as follows.

1. It is assumed that bus overtaking maneuvers are prohibited, both within an entry queue and within the stop itself. Overtaking restrictions of this kind are common in cities, because an overtaking bus can disrupt car traffic in adjacent travel lanes.
2. The bus operating at bays is isolated from the effects of traffic signals.
3. The bus stop system operates in a stable state; the load rate *ρ* = *λ*/*μ* < 1.

### 3.2. Single-Berth Stop

In this section, we firstly analyze and compute the transition probabilities of bus stop system; the balance equations are then formulated and solved for the Markov chain limiting probabilities; and, lastly, the models which are used to calculate the average bus entering delay are proposed.

#### 3.2.1. Transition Probabilities

Firstly, we define the Markov chain transition probabilities:

(2) $$\begin{array}{c} P_{ij}=\mathrm{Pr}\left\{L_{n+1}=j\;|\;L_{n}=i\right\}. \end{array}$$

Let *Y*_*n*_ be the number of buses that arrived in the *n*th cycle, so there is an equation at single-berth bus stop:

(3) $$\begin{array}{c} L_{n+1}=\left\{\begin{array}{cc} Y_{n}, & L_{n}=0,\\ L_{n}+Y_{n}-1, & L_{n}>0. \end{array}\right. \end{array}$$

Let *a*_*j*_ = *P*{*Y*_*n*_ = *j*}, *A*_*j*_ = *P*{*Y*_*n*_ ≤ *j*}; then

(4) P0j=Pr⁡Ln+1=j ∣ Ln=0=PYn=j=aj, j≥0,Pij=Pr⁡Ln+1=j ∣ Ln=i=PYn=j+1−Ln ∣ Ln=i=0,i>j,aj+1−i,i≤j+1.

Let *P* = [*P*_*ij*_, *i* ≥ 0, *j* ≥ 0] be the matrix of transition probabilities; then *P* can be written as follows:

(5) $$\begin{array}{c} P=\left[\begin{array}{c} \begin{array}{cccc} P_{00} & P_{01} & P_{02} & \cdots \\ P_{10} & P_{11} & P_{12} & \cdots \\ P_{20} & P_{21} & P_{22} & \cdots \\ P_{30} & P_{31} & P_{32} & \cdots \\ \cdots & \cdots & \cdots & \cdots \end{array} \end{array}\right]=\left[\begin{array}{c} \begin{array}{cccc} a_{0} & a_{1} & a_{2} & \cdots \\ a_{0} & a_{1} & a_{2} & \cdots \\ 0 & a_{0} & a_{1} & \cdots \\ 0 & 0 & a_{0} & \cdots \\ \cdots & \cdots & \cdots & \cdots \end{array} \end{array}\right]. \end{array}$$

#### 3.2.2. Balance Equation of Limiting Probabilities

**Figure 3:**
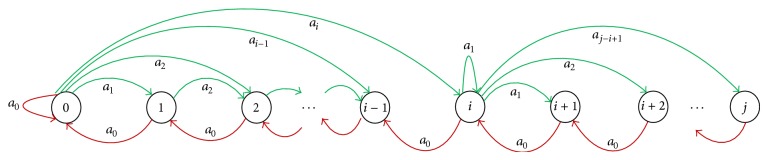
State transition diagram of single-berth bays.

According to (5), the state transition diagram of single-berth bays can be drawn, as shown in Figure 3.

Let *S*_*n*_ be the serving time of the *n*th bus; then {*S*_*n*_, *n* ≥ 0} is a sequence of independent and identically distributed random variables, so we obtain the following equations:

(6) $$\begin{array}{c} a_{j}=P\left\{Y_{n}=j\right\}=\overset{\infty }{\underset{0}{\int }}P\left\{Y_{n}=j\;|\;S_{n}=t\right\}\text{d}F_{S}\left(t\right). \end{array}$$

In (6), *P*{*Y*_*n*_ = *j*∣*S*_*n*_ = *t*} represents the probability that the number of bus arrivals during *t* is *j*. Buses arrived at the stop as a Poisson process, so the equation is

(7) $$\begin{array}{c} P\left\{Y_{n}=j\;|\;S_{n}=t\right\}=\frac{{\left(\lambda t\right)}^{j}e^{-\lambda t}}{j!}. \end{array}$$

Equation (7) was substituted in (6); we have

(8) $$\begin{array}{c} a_{j}=\overset{\infty }{\underset{0}{\int }}\frac{{\left(\lambda t\right)}^{j}e^{-\lambda t}}{j!}\text{d}F_{S}\left(t\right). \end{array}$$

So the expectation of *Y*_*n*_ is computed as follows:

(9) EYn=∑j=0∞jPYn=j=∑j=0∞j·∫0∞λtje−λtj!dFSt  =λ·ESn=ρ,EYn2=∑j=0∞j2PYn=j=ρ+ρ2+λ2DSn.

The variance of *Y*_*n*_ is computed as follows:

(10) $$\begin{array}{c} D\left[S_{n}\right]=E\left[Y_{n}^{2}\right]-E^{2}\left[Y_{n}\right]=\rho +\lambda^{2}D\left[S_{n}\right]. \end{array}$$

Let *π*_*i*_  (*i* ≥ 0) be the limiting probability that the Markov chain is in state *i*; that is, $\pi_{i}=\text{li}{\text{m}}_{n\to \infty }\mathrm{Pr}\{{\tilde{L}}_{n}=i\}$. So *π* = [*π*_1_, *π*_2_,…] represents the limiting distribution of the Markov chain. Thus, *π* is the solution to the balance equation:

(11) $$\begin{array}{c} \pi_{j}=\overset{\infty }{\underset{i=0}{\sum }}\pi_{i}P_{ij}. \end{array}$$

The equations are established according to the characteristics of the generating function, and then the balance equation of single-berth stop is resolved, as follows:

(12) $$\begin{array}{c} \pi \left(u\right)=\frac{\left(1-u\right)\left(1-\rho \right)A\left(u\right)}{A\left(u\right)-u}. \end{array}$$

#### 3.2.3. Average Bus Entering Delay

At the single-berth bus stop, *L*_*n*_ is the number of bus arrivals at the stop during the waiting time *W*_*n*−1_ and service time *S*_*n*−1_ of the (*n* − 1)th bus. Let *F*_*W*_(*t*) and *F*_*S*_(*t*) be the cumulative distribution functions (CDF) of *W*_*n*_ and *S*_*n*_ whichare mutually independent. So the average number of buses in the system can be calculated by the following formula:

(13) N=ELn=∑j=0∞jPLn=j=∑j=0∞∬0∞λt+xjj!e−λt+xdFWtdFSt=∬0∞λt+xdFWtdFSt=λEWn+ESn=λEWq+ρ,

and *N* = *E*[*L*_*n*_] = *π*′(1); (13) was substituted in it; we have

(14) N=π′1=lim⁡u→1ddu1−u1−ρAuAu−u    =ρ+λ2ESn221−ρ.

Combine (13) and (14) to solve the average bus entering delay at single-berth bays:

(15) $$\begin{array}{c} E\left[W_{q}\right]=\frac{\lambda E\left(S_{n}^{2}\right)}{2\left(1-\rho \right)}. \end{array}$$

### 3.3. Two-Berth Bus Stop

The bus entering delay of two-berth bus bays is studied using the same approach as in Section 3.2.

#### 3.3.1. Transition Probabilities

Let *M*_*n*_ be the number of buses that get served in the *n*th cycle. Thus, $\mathrm{Pr}\{{\tilde{L}}_{n+1}=j,M_{n}=k|{\tilde{L}}_{n}=i\}$ for 1 ≤ *k* ≤ 2 represent the probability that the numbers of buses queueing at the stop's entrance at the beginning of the *n*th and (*n* + 1)th cycles are *i* and *j*, respectively, and the number of buses served at the *n*th cycle is *k*.

For an *M*/*G*/2 queueing system, there are six kinds of state transition occurring [14]. We obtain the transition probabilities by finding all the $\mathrm{Pr}\{{\tilde{L}}_{n+1}=j,M_{n}=k|{\tilde{L}}_{n}=i\}$:

(16) P0,0=P1,0=Pr⁡{L~n+1=0,Mn=1 ∣ L~n=0}P1,0=+Pr⁡{L~n+1=0,Mn=2 ∣ L~n=0}=Pr⁡{L~n+1=0,Mn=1 ∣ L~n=1}+Pr⁡{L~n+1=0,Mn=2 ∣ L~n=1},P0,j=P1,j=Pr⁡{L~n+1=j,Mn=2 ∣ L~n=0}P1,j=+Pr⁡{L~n+1=j,Mn=2 ∣ L~n=1}, j>0,Pi,j=Pr⁡{L~n+1=j,Mn=2 ∣ L~n=i}, i≥2,  j≥i−2,Pi,j=0,  else.

Then we determine the expression of each probability in (16) as follows.

(1) $\mathrm{Pr}\{{\tilde{L}}_{n+1}=0,M_{n}=1|{\tilde{L}}_{n}=1\}$: this probability is equivalent to the probability that there is no bus arriving when the first bus finishes its service; thus

(17) Pr⁡{L~n+1=0,Mn=1 ∣ L~n=1} =Pr⁡H1>S1=∫t=0∞e−rtdFSt,

where *H*_1_ is the headway following the first bus arrival in the cycle, s; *S*_1_ is the first bus's service time in the cycle, s.

(2) $\mathrm{Pr}\{{\tilde{L}}_{n+1}=j,M_{n}=2|{\tilde{L}}_{n}=1\}$: through the analysis, there would be at least 2 arrivals in the cycle; that is, *H*_1_ < *S*_1_. Let *τ* be the time between the 2nd arrival and its departure. Because the service time of buses is different, there are two possible scenarios at two-berth bus bays: the first bus finishes its service before the 2nd bus; the 2nd bus finishes its service before the first bus; thus

(18) $$\begin{array}{c} \tau =\max\left\{S_{1}-H_{1},S_{2}\;|\;H_{1}<S_{1}\right\}, \end{array}$$

where *S*_2_ is the second bus's service time in the cycle, s.

We can derive the CDF of *τ* as

(19) Fτt=Pr⁡τ<t=Pr⁡max⁡{S1−H1,S2 ∣ H1<S1}<t=∫h=0∞FSh+t−FShre−rhdhPr⁡H1<S1·FSt.

Thus, for *j* ≥ 0, the probability is computed as follows:

(20) Pr⁡{L~n+1=j,Mn=2 ∣ L~n=1} =Pr⁡H1<S1·∫h=0∞e−rtrtjj!dFτ(t) =∫h=0∞e−rtrtjj!d hhhhh·FSt·∫h=0∞FSh+t−FShre−rhdh.

(3) $\mathrm{Pr}\{{\tilde{L}}_{n+1}=j,M_{n}=2|{\tilde{L}}_{n}=i\}$: according to [6], the CDF of the platoon service time of two buses entering the stop simultaneously is *F*_*S*_^2^(*t*); then for *i* ≥ 2 and *j* ≥ *i* − 2

(21) Pr⁡{L~n+1=j,Mn=2 ∣ L~n=i} =∫h=0∞e−rtrtj−i+2j−i+2!dFS2t.

In summary, the mathematical expectation of transition probabilities of two-berth bus bays is given by

(22) P0,0=P1,0=∫t=0∞e−rtdFSt·1+∫h=0∞FSh+t−FShre−rhdhhhhhhhhll· 1+∫h=0∞FSh+t−FShre−rhdh,P0,j=P1,j=∫h=0∞e−rtrtjj!dhhh·FSt·∫h=0∞FSh+t−FShre−rhdh,hhhhhhhhhhhhhhhhhhhhhhhhhhhhhfor  j>0,Pi,j=∫h=0∞e−rtrtj−i+2j−i+2!dFS2t, for  i≥2,  j≥i−2,Pi,j=0,  else.

#### 3.3.2. Balance Equation of Limiting Probabilities

The solution method of balance equation uses the *z*-transform of *π* to consolidate the infinite-size balance equation into a single functional equation. Then its solution can be converted back to the original distribution:

(23) $$\begin{array}{c} \tilde{\pi }\left(z\right)=\overset{\infty }{\underset{i=0}{\sum }}\pi_{i}z^{i}. \end{array}$$

From the transition probabilities above, the balance equation of limiting probabilities can be written as

(24) $$\begin{array}{c} \pi_{0}P_{0,1}+\pi_{1}P_{1,0}=0,\\ \pi_{n-1}P_{n-1,n}+\pi_{n}\left(P_{n,n+1}+P_{n,n-1}\right)=\pi_{n+1}P_{n+1,n}. \end{array}$$

Then, we have

(25) $$\begin{array}{c} \pi_{k}=\left\{\begin{array}{cc} \overset{\infty }{\underset{j=0}{\sum }}P_{0,j}, & 0\leq k\leq 1,\\ \overset{\infty }{\underset{j=k-2}{\sum }}P_{k,j}, & k>1. \end{array}\right. \end{array}$$

The *z*-transform method is used for this. We can write the balance equation in the *z*-domain as

(26) $$\begin{array}{c} \tilde{\pi }\left(z\right)=\left(\pi_{0}+\pi_{1}\right)\overset{\infty }{\underset{j=0}{\sum }}P_{0,j}z^{i}+\overset{\infty }{\underset{i=2}{\sum }}\pi_{i}\cdot \overset{\infty }{\underset{j=i-2}{\sum }}P_{i,j}z^{j}. \end{array}$$

Let *G*(*t*) = *F*_*S*_(*t*) · ∫_*h*=0_^*∞*^(*F*_*S*_(*h* + *t*) − *F*_*S*_(*h*))*re* ^−*rh*^d*h*; then we have

(27) $$\begin{array}{c} \overset{\infty }{\underset{j=0}{\sum }}P_{0,j}z^{i}\;=\overset{\infty }{\underset{t=0}{\int }}e^{-rt}\text{d}F_{S}\left(t\right)+\overset{\infty }{\underset{t=0}{\int }}e^{rt(z-1)}\text{d}G\left(t\right), \end{array}$$

(28) ∑i=2∞πi·∑j=i−2∞Pi,jzj =∑i=2∞πi·∑j=i−2∞zj∫h=0∞e−rtrtj−i+2j−i+2!dFS2t.

Let *k* = *j* − *i* + 2; (28) can be converted to

(29) ∑i=2∞πi·∑j=i−2∞Pi,jzj =z−2π~z−π0−π1z·∫t=0∞ertz−1dFS2t.

Hence,

(30) π~z=π0+π1∫t=0∞e−rtdFSt+∫t=0∞ertz−1dGthl∫t=0∞e−rtdFSt+∫t=0∞ertz−1dGt−z−2π0+π1z∫t=0∞ertz−1dFS2t·1−z−2·∫t=0∞ertz−1dFS2t−1.

#### 3.3.3. Average Bus Entering Delay

Determining the average bus delay in queue requires the calculation of the average number of buses in queue over time. The average number of buses in queue is equal to the average of the queue length seen by each Poisson bus arrival. So it can be calculated by the next equation:

(31) L−q=lim⁡T→∞∑i=1ATLqiAT=lim⁡T→∞∑i=1ATLqi/NAT/N=lim⁡N→∞∑TLn/Nlim⁡N→∞∑An/N  =T −L−A−,

where ${\overset{-}{L}}_{q}$ is the average number of buses in queue, buses; *L*_*qi*_ is the average of the queue length seen by the *i*th bus arrival during *T*, m; *A*_*T*_ is the number of bus arrivals during *T*, buses; *N* is the number of cycles during *T*; *A*_*n*_ is the number of bus arrivals during cycle *n*, buses; *TL*_*n*_ is the sum of the queue lengths seen by each bus arrival during cycle *n*, m; $\overset{-}{T\;}\overset{-}{L}$ is the average of the sum of the queue lengths seen by each bus arrival during cycle *n*, m; $\overset{-}{A}$ is the average of the number of bus arrivals during each cycle, buses.

To obtain $\overset{-}{T\;}\overset{-}{L}$ and $\overset{-}{A}$, we consider the following four scenarios which describe the possible states of the system at the start and end of each *n*th cycle.

(1) No bus queues are present at the stop's entry, both at the start and at the end of the *n*th cycle; that is, ${\tilde{L}}_{n}={\tilde{L}}_{n+1}=0$. In this scenario, no bus arriving during cycle *n* encounters a queue, and the number of buses that arrive during cycle *n* is the number served during that cycle. So *TL*_*n*_ and *A*_*n*_ can be denoted as
  (32) $$\begin{array}{c} TL_{n}=0,\;\;A_{n}=M_{n}. \end{array}$$
(2) A bus queue is present at the start of cycle *n*, but not at the end of that cycle; that is, ${\tilde{L}}_{n}=i>0$, and ${\tilde{L}}_{n+1}=0$, and *i* < 2. Then *TL*_*n*_ and *A*_*n*_ can be denoted as
  (33) $$\begin{array}{c} TL_{n}=0,\;\;A_{n}=M_{n}-i. \end{array}$$
(3) A bus queue is present both at the start and at the end of cycle *n*, and the number of buses in queue is less than or equal to the number of berths; that is, ${\tilde{L}}_{n}=i\leq 2$, and ${\tilde{L}}_{n+1}=j>0$. In this scenario, the stop is filled during the cycle; that is, *M*_*n*_ = 2, and *j* = *A*_*n*_ + *i* − 2. The first (2 − *i*) arrivals fill unused berths, such that the first (2 − *i* + 1) arrivals see no entry queue. The following arrivals will see successively longer queues that range from 1 to (*j* − 1). Thus,
  (34) $$\begin{array}{c} TL_{n}=\frac{j\left(j-1\right)}{2},\;\;A_{n}=j-i+2. \end{array}$$
(4) A queue size greater than 2 is present at the start of cycle, and a queue thus persists at the end of that cycle; that is, ${\tilde{L}}_{n}=i>2$, and ${\tilde{L}}_{n+1}=j>i-2>0$. In this scenario, as in the previous one, *M*_*n*_ = 2, and *j* = *A*_*n*_ + *i* − 2. And since the earliest bus of cycle *n* is characterized by (*i* − 2) buses that remain in the entry queue, arrivals thereafter will see queue lengths in the sequence (*i* − 2 + 1),…, (*j* − 1). Thus
  (35) $$\begin{array}{c} TL_{n}=\frac{\left(j-2+i\right)\left(j+i-3\right)}{2},\;\;A_{n}=j-i+2. \end{array}$$

Note from the above that the *TL*_*n*_ and *A*_*n*_ only depend on ${\tilde{L}}_{n}$, ${\tilde{L}}_{n+1}$, and *M*_*n*_. Thus, $\overset{-}{T\;}\overset{-}{L}$ and $\overset{-}{A}$ can be obtained by taking weighted averages:

(36) $$\begin{array}{c} \overset{-}{T\;}\overset{-}{L}=\sum_{i,j\cdot k}\mathrm{Pr}\left\{{\tilde{L}}_{n}=i,{\tilde{L}}_{n+1}=j,M_{n}=k\right\}\cdot TL_{n},\\ \overset{-}{A}=\sum_{i,j\cdot k}\mathrm{Pr}\left\{{\tilde{L}}_{n}=i,{\tilde{L}}_{n+1}=j,M_{n}=k\right\}\cdot A_{n}, \end{array}$$

where $\mathrm{Pr}\{{\tilde{L}}_{n}=i,{\tilde{L}}_{n+1}=j,M_{n}=k\}$ are the long-run probability of a cycle where ${\tilde{L}}_{n}=I$, ${\tilde{L}}_{n+1}=j$, and *M*_*n*_ = *k* are calculated by the next equation:

(37) Pr⁡L~n=i,L~n+1=j,Mn=k =πi·Pr⁡⁡L~n=i,Mn=k ∣ L~n+1=j.

So,

(38) T −L−=∑i,j,kPr⁡L~n=i,L~n+1=j,Mn=k·TLn=∑j=0∞π0+π1∫t=0∞e−rtrtjj!dG(t)j(j−1)2+∑i=2∞πi∑j=i−2∞∫t=0∞e−rtrtj−i+2j−i+2!dFS2thhhhhhhhhhhh·i+j−3j−i+22.

Let *k* = *j* − *i* + 2; (38) is converted to

(39) T −L−=π0+π1∫t=0∞rt22dGt+∑i=2∞πi∫t=0∞e−rtrt22+i−2rtdFS2t,A−=∑i,j·kPr⁡L~n=i,L~n+1=j,Mn=k·An= π0∫t=0∞e−rtdFS(t)+π0∫t=0∞(rt+2)dG(t)+π1∫t=0∞rt+1dGt+∑i=2∞πi∫t=0∞rtdFS2t.

Therefore, we have

(40) L−q=π0+π1∫t=0∞rt22dGt∑i=2∞πi∫t=0∞e−rtrt22+(i−2)rtdFS2(t)h+∑i=2∞πi∫t=0∞e−rtrt22+(i−2)rtdFS2(t)·π0∫t=0∞e−rtdFSt+π0∫t=0∞rt+2dGt∑i=2∞πi∫t=0∞rtdFS2(t)hhl+ π1∫t=0∞rt+1dGt+∑i=2∞πi∫t=0∞rtdFS2t−1.

From Little's formula [15], the average bus delay in the queue is then obtained:

(41) Wq=L−qλ=π0+π1∫t=0∞rt22dGt∑i=2∞πi∫t=0∞e−rtrt22+i−2rtdFS2th+ ∑i=2∞πi∫t=0∞e−rtrt22+i−2rtdFS2t·λπ0∫t=0∞e−rtdFSt+π0∫t=0∞rt+2dGt+∑i=2∞πi∫t=0∞rtdFS2thhhhhl+π1∫t=0∞(rt+1)dG(t)hhhhhl+ ∑i=2∞πi∫t=0∞rtdFS2t−1.

The operability of (41) is not strong in practice because it is too complicated. And therefore, it needs to be simplified. The approximate calculation model of bus entering delay of two-berth bays is obtained using the approximation theory of stochastic service system, as follows:

(42) $$\begin{array}{c} W_{q}=\left(0.6C_{S}+3\right){\left(\tan\frac{\pi }{2}\rho \right)}^{\left(0.046C_{S}+1.1\right)}, \end{array}$$

where *C*_*S*_ is the coefficient of variation in bus service time, which is computed by the next equation:

(43) $$\begin{array}{c} C_{S}=\frac{\sigma }{u}=\frac{\sqrt{\left(1/N\right)\sum_{i=0}^{N}{\left(x_{i}-u\right)}^{2}}}{u}. \end{array}$$

## 4. Calculation Models of Bus Exiting Delay

According to the queueing theory and the gap acceptance theory, the average exiting delay is equal to the average number multiplied by the average length of nongaps that bus waits for, as shown in (51). Oliver defined any time interval that is greater than the critical headway as a gap and remaining intervals as nongaps [16]:

(44) $$\begin{array}{c} E\left(t\right)=n_{2}\times {\bar{T}}_{1}, \end{array}$$

where *n*_2_ is the average number of nongaps that bus waits for; ${\bar{T}}_{1}$ is the average length of nongaps.

*(1) Average Number of Nongaps That Bus Waits for*. When headways are assumed to have a negative exponential distribution, the probability that bus will join without delay is

(45) $$\begin{array}{c} p\left(h\geq \tau_{b}\right)=e^{-\lambda_{c}\tau_{b}}, \end{array}$$

where *λ*_*c*_ is the flow at curb lane, pcu/h; *τ*_*b*_ is the critical gap, s.

The probability that bus will be delayed is

(46) $$\begin{array}{c} p_{d}=1-e^{-\lambda_{c}\tau_{b}}. \end{array}$$

The number of blocks is

(47) $$\begin{array}{c} n=\lambda_{c}Te^{-\lambda_{c}\tau_{b}}. \end{array}$$

The average number of vehicles between the starts of gaps is

(48) $$\begin{array}{c} n_{1}=\frac{1}{e^{-\lambda_{c}\tau_{b}}}. \end{array}$$

Therefore, the average number of nongaps that bus waits for is

(49) $$\begin{array}{c} n_{2}=\frac{1}{e^{-\lambda_{c}\tau_{b}}}-1. \end{array}$$

*(2) Average Length of Nongaps*. The total time spent in the nongaps is

(50) T1=T−∫τb∞λcTe−λctdt=T−λcT−1λce−λctτb∞    =T×1+e−λcτb.

The total number of nongaps is

(51) $$\begin{array}{c} n_{3}=\lambda_{c}T\left(1-e^{-\lambda_{c}\tau_{b}}\right). \end{array}$$

The average length of nongaps is

(52) $$\begin{array}{c} {\bar{T}}_{1}=\frac{T_{1}}{\lambda_{c}T\left(1-e^{-\lambda_{c}\tau_{b}}\right)}=\frac{1+e^{-\lambda_{c}\tau_{b}}}{\lambda_{c}\left(1-e^{-\lambda_{c}\tau_{b}}\right)}. \end{array}$$

From this, it is noted that the average exiting delay is found by multiplying the average number by the average length of nongaps that bus waits for; that is,

(53) Wb=Et=1e−λcτb−11+e−λcτbλc1−e−λcτb=1+e−λcτbλce−λcτb.

## 5. Calculated Results

### 5.1. Single-Berth Bay

Based on the above analysis, the average bus delay at single-berth bays is calculated by (54):

(54) $$\begin{array}{c} W=\frac{\lambda E\left(S_{n}^{2}\right)}{2\left(1-\rho \right)}+\frac{\lambda -q_{s}e^{-\lambda \tau_{b}}}{\lambda q_{s}e^{-\lambda \tau_{b}}}. \end{array}$$

**Figure 4:**
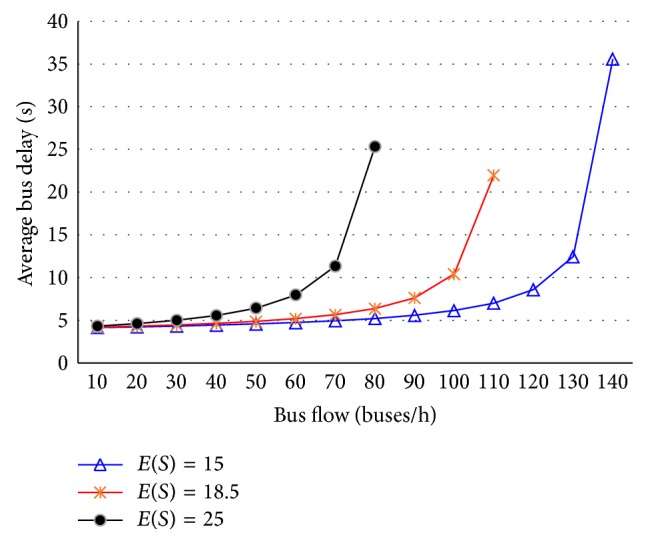
The average bus delay at single-berth bays.

The average bus delay is calculated with (54) under different demands at single-berth bays, as shown in Figure 4. It can be seen that the bus delay grows slowly when bus flow is less than 60 buses/h and has a rapid growth once bus flow exceeds 60 buses/h.

### 5.2. Two-Berth Bay

The average bus delay at two-berth bays is calculated by

(55) W=0.6CS+0.3tanπ2ρ0.046CS+1.1=+λqs−e−λτbλe−λτb.

**Figure 5:**
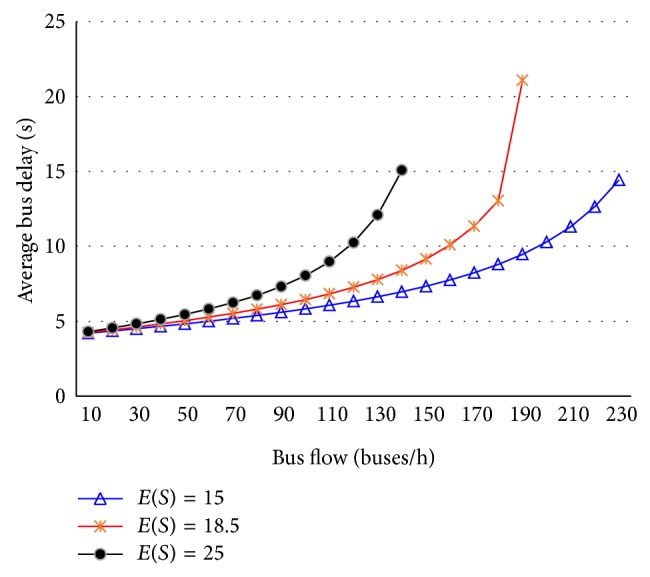
The average bus delay at two-berth bays.

The average bus delays are calculated with (55) under different demand at two-berth bays, as shown in Figure 5. It can be seen that the bus delay grows slowly when bus flow is less than 100 buses/h and has a rapid growth once bus flow exceeds 100 buses/h.

## 6. Model Validation

The proposed model is validated using measured data at two bus bays of Tianmushan Road in Hangzhou city. The arriving time, queueing length, and service time of buses at Jingzhou North Intersection and Gudun Intersection bays during peak hours are surveyed by video. Then the delay of every bus is obtained by processing these data, as shown in Table 1. It can be seen that the average relative error between the calculated and the measured values of the bus delay is 10.44%. It shows that the proposed model can effectively reflect the operating characteristics of bus bays.

## 7. Influencing Factors Analysis

**Figure 6:**
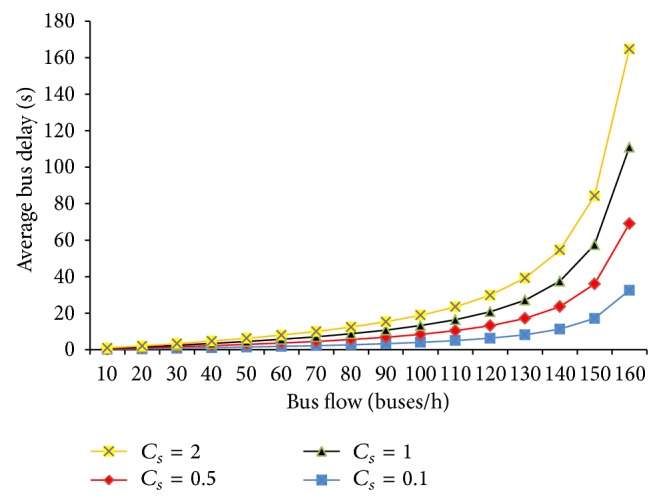
Impact of coefficient of variation in bus service time on bus delay.

The calculated results above show that the bus delay depends mainly on the average service time at given bus bays. The longer the average service time, the smaller the capacity of stop, which means the bus delay will increase. In addition, it is also affected by the coefficient of variation in bus service time at multiberth bus bays, and the impact characteristics are shown in Figure 6. Visual inspection of this figure reveals that the value of bus delay grows larger with growing *C*_*S*_ in the same bus flow. This is mainly due to the increasing impact between the servicing buses. Therefore, too many bus lines should not be set on the same stretch of road. Because more bus lines will surely increase the coefficient of variation in bus service time, the interaction between buses will be intensified. According to previous experience, 4 or 5 bus lines at most are set up on the major roads in the city [4, 5].

## 8. Conclusion

Formulas were developed to predict the average bus delay at bays. The formulas use a Markov chain that is embedded in the bus queueing process, the queueing theory, and the gap acceptance theory at these bays. Exact solutions were derived for two special cases: single-berth and two-berth bays. And approximations matched up to the surveyed results. With this methodology, the bus delays at bays are obtained easily if the characteristics of the service time distribution and traffic flow are known. And the results of this paper can provide basis for the efficiency evaluation of bus bays.

## Figures and Tables

**Table 1:** Comparison of the calculated and measured values of bus delay at bays.

Bus bay	Number of berths	Distribution of dwell time (*μ*, *σ* ^2^)	Bus flow (buses/h)	Car flow at curb lane (veh/h)	Calculated values (s)	Measured values (s)	Relative error (%)
Jingzhou North Intersection	2	(2.856, 0.325)	96	360	5.68	5.12	9.86
Gudun Intersection	2	(2.931, 0.414)	120	420	10.52	11.68	11.03
Average	—	—	—	—	—	—	**10.44**
